# Metabolic changes and potential biomarkers in "*Candidatus* Liberibacter solanacearum"-infected potato psyllids: implications for psyllid-pathogen interactions

**DOI:** 10.3389/fpls.2023.1204305

**Published:** 2023-07-19

**Authors:** Yelin Li, Zhiqing Tan, Xiaolan Wang, Liping Hou

**Affiliations:** ^1^ School of Life Sciences, Guangzhou University, Guangzhou, China; ^2^ School of Life Sciences, Zhaoqing University, Zhaoqing, China; ^3^ Guangdong Provincial Key Laboratory of Plant Adaptation and Molecular Design, Guangzhou University, Guangzhou, China

**Keywords:** infection, psyllid, metabolism, pathogen, interaction, potential biomarkers

## Abstract

Psyllid yellows, vein-greening (VG), and zebra chip (ZC) diseases, which are primarily transmitted by potato psyllid (PoP) carrying *Candidatus* Liberibacter solanacearum (*C*Lso), have caused significant losses in solanaceous crop production worldwide. Pathogens interact with their vectors at the organic and cellular levels, while the potential changes that may occur at the biochemical level are less well reported. In this study, the impact of *C*Lso on the metabolism of PoP and the identification of biomarkers from infected psyllids were examined. Using ultra-performance liquid chromatography tandem mass spectrometry (UPLC-MS/MS) analysis, metabolomic changes in *C*Lso-infected psyllids were compared to uninfected ones. A total of 34 metabolites were identified as potential biomarkers of *C*Lso infection, which were primarily related to amino acid, carbohydrate, and lipid metabolism. The significant increase in glycerophospholipids is thought to be associated with *C*Lso evading the insect vector’s immune defense. Matrix-assisted Laser Desorption Ionization Mass Spectrometry Imaging (MALDI-MSI) was used to map the spatial distribution of these biomarkers, revealing that 15-keto-Prostaglandin E2 and alpha-D-Glucose were highly expressed in the abdomen of uninfected psyllids but down-regulated in infected psyllids. It is speculated that this down-regulation may be due to *C*Lso evading surveillance by immune suppression in the PoP midgut. Overall, valuable biochemical information was provided, a theoretical basis for a better understanding of psyllid-pathogen interactions was offered, and the findings may aid in breaking the transmission cycle of these diseases.

## Introduction

The potato psyllid (PoP), *Bactericera cockerelli* (Sulc) (Hemiptera : Triozidae), is a phloem-feeding member of the Hemiptera family commonly found on plant species of the Convolvulaceae and Solanaceae families (Baumann, 2005; Fisher et al., 2014). As the vector of the unculturable gram-negative *C*Lso, once it feeds on potato or tomato plants, the psyllid transmits *C*Lso through its saliva, causing psyllid yellows, vein-greening (VG) and zebra chip (ZC) diseases to occur (Alvarado et al., 2012; Mishra and Ghanim, 2022). The common symptoms of psyllid yellows include yellowing of leaves, aerial tubers, shortened and thickened internodes, stunted plant growth, and in extreme cases caused plant death (Munyaneza et al., 2007; Goolsby et al., 2012). It has been reported to cause a decrease in potato crop yield and quality, and ZC disease could result in potato yield losses up to 94% (Greenway, 2014). *C*Lso-infected psyllids can also lead to severe economic losses, in Texas, ca. 33 million dollars annually in potato production due to ZC disease (Prager et al., 2022).

PoP is the only known carrier of the *C*Lso that causes ZC disease (Rondon et al., 2022). Due to the lack of effective integrated management approach for ZC, currently, controlling the spread of ZC and VG diseases caused by *C*Lso is achieved managing the psyllid vector by the use of chemical pesticides, which is costly and poses risks to the environment and human health (Mora et al., 2021). One potential solution to this problem is identifying and deploying *C*Lso-resistant varieties. This is an effective long-term pest management strategy, but it can take a significant amount of time to develop these varieties, and there is the possibility that the pressure of evolution may lead to the development of resistant PoP varieties. Therefore, a more comprehensive and sustainable management plan is needed that not only incorporates genetic tools but also a deeper understanding of the interactions between *C*Lso, its plant hosts, and its insect vector.

Multiple studies have investigated the interactions between psyllids and the bacteria they transmit. For instance, research on the Asian citrus psyllid (ACP) has revealed that infection with *Candidatus* Liberibacter asiaticus (*C*Las) leads to a down-regulation of the phenoloxidase enzyme. Phenoloxidase is associated with the melanization defense response, suggesting that *C*Las suppresses the immune system of the ACP (Eleftherianos and Revenis, 2011). Additionally, the expression of the hexamine protein was found to be suppressed in *C*Las-infected adults, which could indicate that *C*Las may regulate the availability of free amino acids by interfering with hexamerin storage pathways (Burmester et al., 1998).

Transcriptome analysis of infected psyllids has also shown that *C*Lso greatly affects genes involved in metabolic processes, while having a lesser effect on genes associated with immune and stress response (Nachappa et al., 2012). Furthermore, studies of genes related to metabolism and nutrition of adult PoP have revealed that *C*Lso infection alters purine, carbon, pyrimidine, glycerophospholipid, and choline metabolism (Vyas et al., 2015). These studies demonstrate that the bacteria transmitted by psyllids has significant impacts on the physiology, biochemistry, and immunity of the psyllids themselves as well.

Recent studies have also lucubrated the metabolic changes of the ACP when it is infected with the bacteria *C*Las. Research has found that the levels of adenosine triphosphate (ATP) in *C*Las-infected psyllids were higher than in uninfected psyllids, indicating that *C*Las altered the energy metabolism, which means *C*Las took up ATP by using ATP translocase of its psyllid vector (Killiny et al., 2017). Additionally, it has been reported that *C*Las infection can elicit a response to biotic stress or cell damage, and induce nutrient and energetic stress in the host insect (Killiny et al., 2017). These studies have provided a deeper understanding of the metabolic changes that occur in this important insect pest and disease vector at the metabolomic level. As *Liberibacter* species act as intracellular parasites, understanding the metabolic connections between the bacteria and their hosts is a crucial aspect of disease management.

However, the metabolic effects of *C*Lso-infection on its host, the potato or tomato psyllid, have not yet been fully explored. Understanding how metabolic pathways within the potato psyllid host are affected or respond to infection by *Liberibacter* species is crucial for identifying more effective disease management strategies (Pratavieira et al., 2014; Cicalini et al., 2019; Yang et al., 2020).

UPLC-MS/MS, as a high-throughput assay for the rapid detection and identification of metabolites, has been applied to several insect models, such as *Drosophila* (Tuthill et al., 2020), Africanized honey bees (*Apis mellifera* L.) (Barbosa-Medina et al., 2022) and *Aedes* mosquitoes (Chen et al., 2020). Matrix-assisted Laser Desorption Ionization Mass Spectrometry Imaging (MALDI-MSI) not only highly sensitive, but also enables visualization and localization of metabolites in tissues and insect organisms (Susniak et al., 2020; Tuck et al., 2022). The use of MALDI-MSI in combination with ultra performance liquid chromatography (UPLC) has been shown to be effective technique, such as identification of lipid species and tissue-specific analysis in high-sugar-fed *Drosophila* (Tuthill et al., 2020).

In this study, the goal was to use this technology to investigate the changes in PoP metabolites upon infection with the bacterium *C*Lso, with the aim of identifying biomarkers for quick identification of infected psyllids. The changes in these metabolites, whether an increase or decrease, may have influenced the interaction between *C*Lso and the psyllid host and may have facilitated further psyllid transmission to plants. Therefore, the analysis of metabonomic changes between *C*Lso-infected and uninfected psyllids may provides more information about how PoPs respond to and cope with *C*Lso infection.

## Materials and methods

### Chemicals

Acetonitrile and formic acid (Fisher Scientific, Loughborough, UK) of HPLC-grade were used. Ultrapure water was produced using a Milli-Q plus (Milford, MA, USA) water purification system. Methanol was supplied by Merck (Darmstadt, Germany). Leucine enkephalin was obtained from Waters (Milford, USA). Organic matrix compound, 2,5-dihydroxybenzoic acid (DHB, 98%) was purchased from Sigma-Aldrich (St. Louis, MO, USA). Gelatin from porcine skin (300 bloom) was purchased from Electron Microscopy Sciences (Hatfield, PA, USA).

### Insect source

Live adult psyllids (*Bactericera cockerelli*) (Sulc) that were infected or uninfected with *C*Lso were collected from the potato/tomato psyllid colony maintained by Dr. Brown’s lab at the University of Arizona as described (Fisher et al., 2014). The cytochrome oxidase I gene as a molecular marker to haplotype psyllid as “central type”, and primers were used to amplify 16S rRNA gene to routinely detect the presence of *C*Lso in colonies by PCR. The collected live psyllids were flash frozen in liquid nitrogen and stored at -80°C until further processed. A total of 100 *C*Lso-infected and 100 uninfected PoP mature adults were selected and divided into 5 sets per treatment type evenly, with 20 PoP in each set. Each set comprised a separate biological replicate.

### Metabolite extraction

The extraction of metabolites for UPLC-MS/MS analysis was conducted as described in Overgaard et al. (Overgaard et al., 2007) with minor modifications. In brief, 20 psyllids were randomly selected from each of the infected and uninfected groups. The samples were pulverized using a TissueLyser II (Qiagen) in precooled (liquid nitrogen) 1.5 mL microfuge tubes using a steel ball mill by operating the instrument as follows: running for 30 seconds, stopping for 30 seconds, and repeating this cycle three times at 30 Hz. Next, 400 μL 50% methonal was added and the samples were vortexed briefly, followed by sonication for 5 minutes with the tubes partially submerged in a water bath at room temperature (5200 Bransonic, Danbury, CT, USA). After centrifugation at 20,000 × g (22,000 *rpm*) for 5 minutes, the methonal was transferred to a fresh microfuge tube. The pellet was resuspended in an additional 400 μL of 50% methonal, sonicated, and centrifuged again for 5 min, respectively, and the methonal was combined with the first sample extraction. The pellets were then extracted in the same manner, but using ethyl acetate as solvent. The three ethyl acetate fractions for each sample were combined with the mixture. After three sequential extractions, a centrifugal evaporator (CentriVap Cold Traps, Labconco) was used for solvent removal and sample dryness. Then, the dried extracts were dissolved in 100 μL of methanol/water solution (1:1) and centrifuged at 20,000 × g for 15 min at 4°C. 90 μL supernatants were analyzed *via* LC-MS. All samples of the same volume to be measured were mixed as quality control (QC) samples to monitor the stability of the analysis.

### UPLC-MS/MS analysis

Samples were analyzed using a Waters ACQUITY UPLC system (Waters Corporation in Milford, USA) that was coupled with a Waters Synapt G2-S HDMS ion mobility quadrupole time-of-flight mass spectrometry (IM-Q-TOF-MS). The injection volume for each sample was 2 μL. Chromatographic separations were achieved using an ACQUITY BEH C18 column ((100 mm × 2.1 mm, particle size1.7 μm; Waters). The column was eluted at a flow rate of 400 μL·min^-1^, using mobile phase A consisting of 0.1% formic acid in HPLC-grade water, and mobile phase B consisting of 0.1% formic acid in methanol. An elution gradient system was used, starting with 85% of mobile phase A and 15% of mobile phase B for 1.5 minutes, before it was gradually changed over 6 minutes to 65% A and 35% B, then another 2 minutes to 50% A and 50% B, then in 1 minute to 20% A and 80% B, and maintained at this level for another 1 minute before returning to the initial conditions of 85% A and 15% B over 0.5 minutes. The column was then re-equilibrated at 85% A for 4 minutes. The total analysis time for each sample was 14 minutes. Five biological replicates were performed for each of the two treatment types.

The separated components were analyzed using a Waters Synapt G2-S HDMS ion mobility quadrupole time-of-flight mass spectrometry (IM-Q-TOF-MS) equipped with an ESI-ion mode. The detection parameters were set as follows: capillary voltage of 3 kV, cone voltage of 30 V, calibration with sodium formate, and high resolution mode for the TOF resolution. A lock-mass of leucine encephalin (200 pg/mL) was employed as a lock spray, and a blank was analyzed between every two samples to clean the column. The mass spectrometric profiles were acquired from a scan mass range of 100 to 1000 *m/z* in positive and negative ionization mode.

### Data analysis

We performed peak picking, alignment, and normalization of the mass spectral data obtained by UPLC-MS/MS using the ProgenesisQI software program (Waters Corporation, Milford, USA). Based on the retention time [RT], mass-to-charge ratio [*m/z*], and peak intensity from the untargeted analysis, data were exported to Ezinfo and Metlin to identify specific ions that differed significantly in abundance between uninfected and infected insects. The resulting UPLC-MS/MS data, including peak numbers, sample names, and normalized peak areas, were inputted into the SIMCA 14.1 software package (Umetrics, Umea, Sweden) for principal component analysis (PCA) and supervised orthogonal projection to latent structures discriminate analysis (OPLS-DA) models, which were used to evaluate the relationships between the *C*Lso-infected and uninfected samples. The differential metabolites were identified according to the variable importance in projection (VIP) values >1.0 obtained from the OPLS-DA model and p-values <0.05 obtained by Student’s t-test, then Kyoto Encyclopedia of Genes and Genomes (KEGG) and LIPID MAPS Structure Database (LMSD) were used for further screening of differential metabolites. KEGG (https://www.genome.jp/kegg/) was used to analyze the enrichment of metabolites, and the enriched metabolic pathways were subjected to a heatmap analysis of multiple change. The KEGG pathways analysis and visualization were done using the free web-based tools, MetaboAnalyst 5.0 (https://www.metaboanalyst.ca/).

### Tissue section preparation

Frozen psyllids were placed in a 30% w/v sucrose solution for 1 hour of sucrose infiltration. The insects were then embedded in a 10% w/v gelatin block by laying them out in the same orientation on a pre-made gelatin block and covering them with molten gelatin cooled just before it re-sets. The finished block was trimmed to remove excess gelatin, mounted on a sample holder using molten gelatin, and then frozen. The solidified sample blocks were cut into 20 µm thick sections using a -23°C Leica CM1950 cryostat (Leica Biosystems GmbH, Nussloch, Germany). The frozen tissue slices (serial sections) were then thaw-mounted on standard non-coated histological glass slides (76 mm × 26 mm × 1.2 mm) for MALDI-MSI analysis. About 10 sections were placed on a single sample slide and were desiccated before the matrix application.

### MALDI-MSI

Insect samples for MALDI-MSI analysis were prepared using a method similar to the one previously described by Niehoff et al. (Niehoff et al., 2014), with some modifications. 2,5-DHB (20 mg·mL^-1^) dissolved in 50% methanol was chosen as the matrix material and was sprayed onto the tissue slices using an automated sprayer (TM-Sprayer™, HTX Technologies, Carrboro, NC, USA) at a pressure of 80 bar (N) and 16 cycles. After matrix application, the slides were dried at room temperature for 1 hour and then mounted on a sample plate designed for glass slides.

MSI analysis was conducted in positive ion mode using a MALDI-solariX 9.4T FTICR-MS System (Bruker Daltonics Inc, Billerica, USA) equipped with a Smartbeam II laser. The FTMS Control 3.0 and FlexImaging 3.2 software packages (Bruker Daltonics, Bremen, Germany) were used to control the mass spectrometer and set imaging parameters (1 kHz laser, medium spot size laser, 350 ns pulsed ion extraction, mass range of *m/z* 100-2,000, 300 laser shots per spot) and data analyzed by FlexImaging and ClinproTools software (Bruker Daltonics Inc).

For images shown, the *C*Lso infected and uninfected sections were imaged on one plate. Total ion current normalization calculates the normalized peak intensity for each spectral feature (peak) by dividing its intensity by the total ion current in each mass spectrum across an entire imaging data set, in this case across four lens images. Ion intensities are then displayed across the image by assigning the largest normalized signal for each *m/z* to a value of 100% and the lowest normalized signal as 0%. Therefore, each ion image is calculated independently from other ion images. For display purposes, data were interpolated and pixel intensities were rescaled using “brightness optimization” in FlexImaging software. This feature optimizes the brightness of each individual signal to use the entire dynamic range, as indicated by the color scale bars. The integrated intensity for each *m/z* signal (± 0.001% *m/z* units) was plotted as a normalized TIC value. A total of three technical replicates were run to ensure reproducibility of molecular patterns.

## Results

### Overall changes in metabolites of PoP after *C*Lso infection

**Figure 1:**
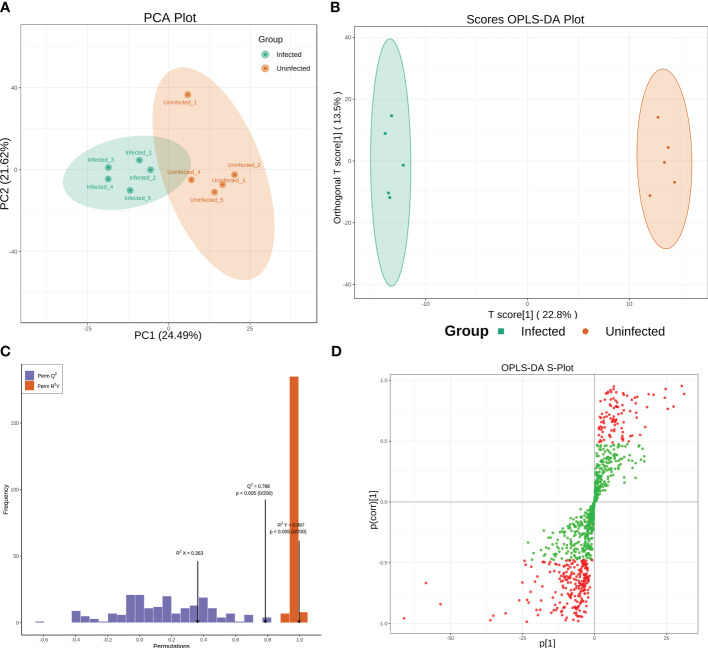
**(A, B)** Principal Component Analysis (PCA) score plot and OPLS-DA scores plot of infected and uninfected group. Each point in the figure represents a sample, and the same set of samples was represented using the same color. **(C)** Permutation tests of the OPLS-DA model. **(D)** S-plot of the OPLS-DA model. Red dots indicated that the VIP values of the metabolites ≥ 1 and green dots indicated that the VIP values of the metabolites ≤ 1.

Metabolomic analyses were conducted using a UPLC-MS-based method. To classify groups suspected of showing metabolic differences, PCA and OPLS-DA approaches, which are commonly used in metabolomics, were employed. PCA shown a separation between infected and uninfected PoP (**Figure 1A**). The separated different clusters indicated that *C*Lso-infection changed the metabolic profiles of PoP. To further differentiate the metabolite features and identify potential marker metabolites, a supervised multivariate data analysis approach, OPLS-DA, was performed. As shown in **Figure 1B**, the infected group was clearly separated from the uninfected group. To ensure the model’s quality, the permutation testing of OPLS-DA model was performed. The cumulative values of R^2^Y and Q^2^ is 0.997 and 0.786, respectively, suggesting that the OPLS-DA model had good fitting and high predictability (**Figure 1C**) and could be used for further analysis. The S-plot was used to show the differentially accumulated metabolites (DAMs) between infected and uninfected groups (**Figure 1D**), which may be regarded as potential biomarkers.

### Identification of potential biomarkers

**Figure 2:**
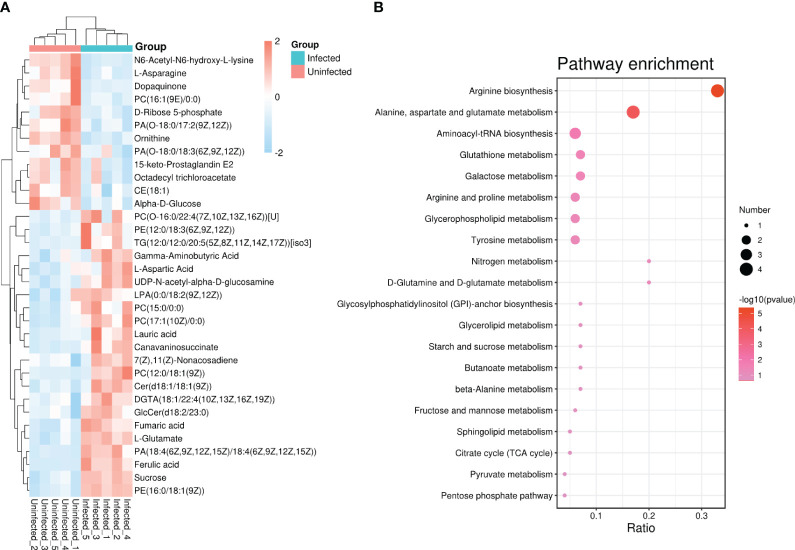
**(A)** Heat map analysis of 34 differential metabolites between infected and uninfected psyllids. The values of differential metabolites were shown as a color scale; **(B)** Results of KEGG pathway enrichment analysis of differential metabolites. The x axis indicates that the proportion and numbers of metabolites annotated to the pathway, and the y axis indicates the name of KEGG metabolic pathway. Dot color and dot size represent the P-value and the number of DAMs respectively.

Based on the goal for exploring potential biomarkers, so VIP ≥ 1 and p < 0.05 as a screened criteria, a total of 142 metabolites were identified (**Supplementary Table S1**). To further target markers associated with pathogen infection, KEGG database and LMSD database were used. Among 142 metabolites, 34 metabolites were matched to the known substances. They included 19 lipids and lipid-like molecules, 9 organic acids and derivatives, 3 other organic compounds, 1 phenylpropanoids and polyketides, and 2 other compounds (**Supplementary Table S2**). To clearly visualize the variation trends in metabolite between infected and uninfected groups, a heat map was drawn (**Figure 2A**). Compared to the control group, 22 metabolites were up-regulated (shown in red) and 12 metabolites were down-regulated (shown in blue) in the infected group (**Supplementary Table S3**).

### Metabolic pathway analysis of infected and uninfected PoP

To identify the most relevant metabolic pathways involved in *C*Lso infection, KEGG enrichment analysis was employed. The x axis indicates that the proportion and numbers of metabolites annotated to the pathway, and the y axis indicates the name of KEGG metabolic pathway. The P-value calculated from the pathway topology analysis was set to 0.05. *C*Lso infection significantly affected arginine biosynthesis, alanine, aspartate, and glutamate metabolism, glutathione metabolism and galactose metabolism as shown in **Figure 2B**.

**Figure 3:**
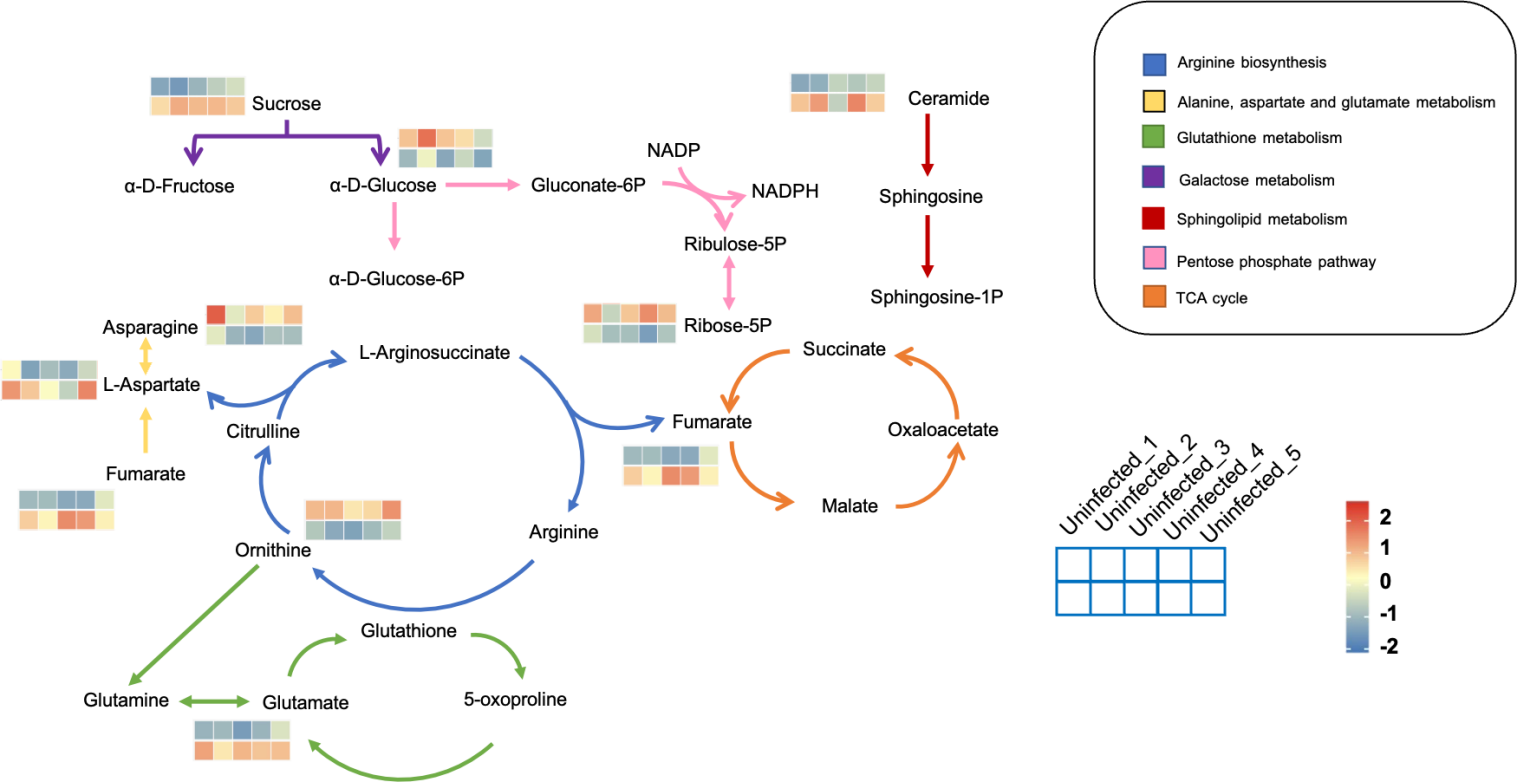
The metabolic framework based on the DAMs. Different metabolic pathways were marked with arrows of different colors and the corresponding color of each metabolic pathway is shown in the legend. For example, the arginine synthesis pathway was marked in blue. The changes in metabolites were represented by 10-small-squares heat map, with the top five squares representing the five biological replicates from uninfected groups and the bottom five squares representing the five biological replicates from infected groups. Colors correspond to the significance of accumulated changes of DAMs. Blue: down-regulated compared with uninfected groups. Red: up-regulated compared with uninfected groups.

The metabolic framework based on the DAMs in the infected PoP compared to the uninfected was shown in **Figure 3**. In infected psyllids, the content of sucrose was up-regulated but the α-D-Glucose was down-regulated, suggesting that *C*Lso affected the galactose metabolism of PoP. The reduction of α-D-Glucose, which is the source of pentose phosphate pathway, leaded to a decrease of its metabolite Ribose-5P. L-aspartic acid, which can enter arginine biosynthesis and affect the content of ornithine, is increased by fumarate through the alanine-aspartate-glutamate metabolism. It indicated that L-aspartic acid as a key metabolite affected the metabolism of *C*Lso-infected PoP. Although there was little effect on most of intermediates in glutathione metabolism, the content of glutamate increased significantly after *C*Lso-infection.

### MALDI-MSI analysis

**Figure 4:**
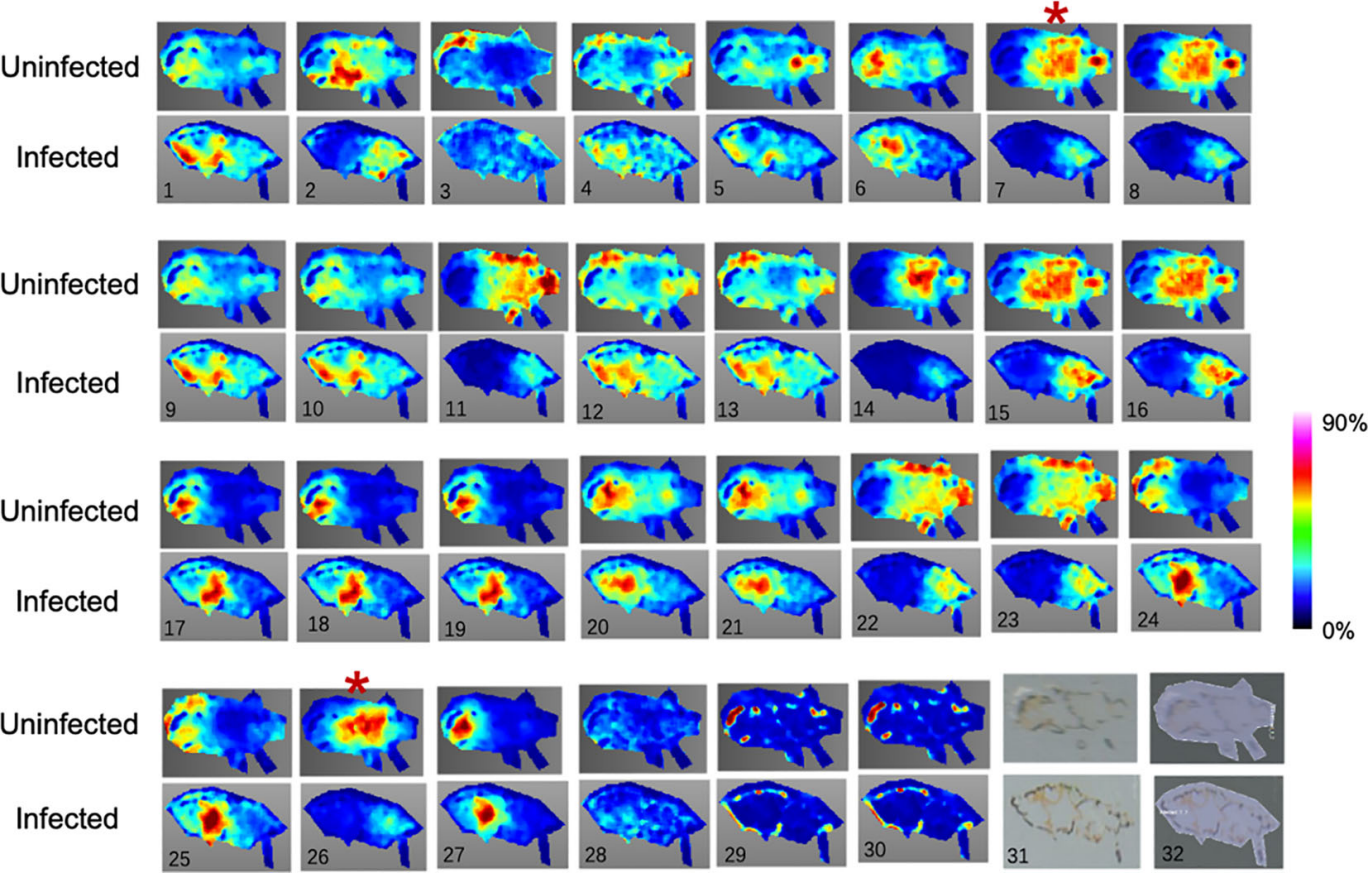
The MALDI-MSI and LC-MS analysis of the 30 metabolites differentially expressed between uninfected and infected psyllid. No.31 is the photograph of insects under a light microscope, and No.32 is the photomirror image of insects with DHB matrix. The 30 metabolites in the image correspond to the order in **Table 1**.

To identify the spatial location of potential biomarkers in PoP tissues, the distribution of these potential biomarkers was examined in psyllid using MALDI-MSI. In a single slice of psyllid, the spatial location images of 30 potential biomarkers were obtained, but 4 of them were not detected in this slice (**Table 1**). Among them, CE(18:1), 15-keto-Prostaglandin E2, Ornithine, PA(O-18:0/18:3(6Z,9Z,12Z)), PC(16:1(9E)/0:0), Dopaquinone, D-Ribose 5P, Octadecyl trichloroacetate, and α-D-Glucose were mainly expressed in the head and abdomen of uninfected PoP, but not in infected **(** **Figure 4**, 7, 8, 14, 15, 16, 22, 23, 26); Ferulic acid, TG (12:0/12:0/20:5(5Z,8Z,11Z,14Z,17Z)), PC(12:0/18:1(9Z)), PA(O-18:0/17:2(9Z,12Z)), and Sucrose were clearly visible behind the abdomen in infected (**Figure 4**, 1, 17, 18, 24, 25). This result indicated that MALDI-MS Imaging and LC-MS analysis provide complementary data sets for biomarkers identification. Interestingly, the metabolites 15-keto-Prostaglandin E2 and alpha D-Glucose were significantly down-regulated in the adbomen of infected psyllids. Combining these results, it can be concluded that the metabolites 15-keto-Prostaglandin E2 and α-D-Glucose can potentially act as candidate biomarkers for *C*Lso infection.

**Table 1:** 30 potential biomarkers.

No.	VIP	Average Mz	Average RT(min)	identified results	FC
1	1.95	416.14	0.323	Ferulic acid	9.21
2	1.41	433.23	6.6	LPA(0:0/18:2(9Z,12Z))	1.52
3	1.47	685.57	10.926	CE(18:1)	0.48
4	1.48	132.03	0.338	L-Aspartic Acid	1.80
5	1.94	146.04	0.338	L-Glutamate	1.65
6	1.40	102.05	0.336	γ -Aminobutyric Acid	1.55
7	1.39	385.18	5.144	15-keto-Prostaglandin E2(PGE2)	0.56
8	1.96	131.08	0.313	Ornithine	0.54
9	1.93	723.37	2.787	PA(18:4(6Z,9Z,12Z,15Z)/18:4(6Z,9Z,12Z,15Z))	13.61
10	1.85	644.03	0.375	UDP-N-acetyl-alpha-D-glucosamine	3.06
11	1.92	241.09	0.311	N6-Acetyl-N6-hydroxy-L-lysine	0.53
12	1.99	719.20	0.34	PE(16:0/18:1(9Z))	2.72
13	1.84	115.00	0.352	Fumaric acid	2.71
14	1.36	719.48	11.224	PA(O-18:0/18:3(6Z,9Z,12Z))	0.54
15	1.42	492.31	7.22	PC(16:1(9E)/0:0)	0.47
16	1.51	230.02	0.35	Dopaquinone	0.17
17	1.46	775.57	11.184	TG(12:0/12:0/20:5(5Z,8Z,11Z,14Z,17Z))[iso3]	10.18
18	1.58	738.50	9.867	PC(12:0/18:1(9Z))	6.04
19	1.46	693.31	1.62	PE(12:0/18:3(6Z,9Z,12Z))	3.60
20	1.40	830.59	11.225	PC(O-16:0/22:4(7Z,10Z,13Z,16Z))[U]	3.14
21	1.69	601.14	0.352	Cer(d18:1/18:1(9Z))	2.39
22	1.71	264.99	0.356	D-Ribose 5-phosphate	0.48
23	1.39	449.15	5.413	Octadecyl trichloroacetate	0.54
24	1.60	672.99	0.379	PA(O-18:0/17:2(9Z,12Z))	0.50
25	1.94	377.08	0.338	Sucrose	1.87
26	1.62	215.03	0.334	α-D-Glucose	0.51
27	1.55	480.30	7.795	PC(15:0/0:0)	1.59
28	1.69	167.02	0.352	L-Asparagine	0.58
29	1.36	439.41	10.66	7(Z),11(Z)-Nonacosadiene	1.85
30	1.61	830.63	11.191	GlcCer(d18:2/23:0)	2.00

## Discussion

*C*Lso infection has a significant impact on its host plants and insects, resulting in diseases such as psyllid yellows, vein-greening (VG), and zebra chip (ZC) (Cevallos-Cevallos et al., 2012; Tamborindeguy et al., 2017). Additionally, the *C*Lso pathogen significantly alters the primary and secondary metabolites in its host insects.

Compared to uninfected insects, the contents of α-D-Glucose and Ribose-5P were significantly decreased, but sucrose was increased in infected insects (**Figure 3**). When PoP is infected, the *C*Lso competes with it for carbohydrates and uses the energy produced by the host plant to meet its own metabolic needs, such as growth and reproduction. The decrease in glucose content may result from its consumption by *C*Lso. It has been reported that the ATP level of *C*Las-infected psyllids *in vivo* is significantly higher than that of healthy psyllids, and the salivation time is reduced, indicating that the *C*Las-infected psyllids experience a higher level of hunger and have a tendency to forage more. The increase in feeding activity reflects the energy stress caused by *C*Lso infection (Killiny et al., 2017). On the other hand, during infection, the activation of cellular immunity leads to a metabolic switch within the immune cells, which become dependent on a massive supply of glucose and glutamine (Dolezal et al., 2019). Generally, the *C*Lso manipulates the energy metabolism of its insect vectors to ensure its need for high-energy nucleotides. As a result, the glucose requirement of the host plant increases sharply, and the psyllid balances its carbohydrate requirement by sucking more sucrose from the phloem of its host plant. The levels of many amino acids were also altered in the host plant by *C*Lso infection. For example, L-aspartic acid, L-glutamate, and fumarate were significantly increased. It has been reported that amino acids act as the main energy source and help organisms synthesize immune effectors to participate in the immune priming response (Wu et al., 2022).

In addition, the level of GABA in *C*Lso-infected PoP was higher than that of the controls. GABA is a free amino acid that plays an important role as a signaling molecule during plant stress. Previous studies have shown that when plants are subjected to abiotic stress such as salt, drought, and high temperature, as well as biological stress such as pathogen infection and insect feeding, GABA levels increase sharply to defend against such stress. Additionally, GABA is also necessary for the growth of pests (Ramesh et al., 2017). Our hypothesis is that the increased GABA content in psyllid may be related to feeding on infected host plants. On the other hand, the level of GABA was also higher in PoP after *C*Lso infection, probably because of the increased glutamate content. Glutamic acid can be converted to GABA through the action of GAD (glutamic acid decarboxylase), and the increase of L-glutamic acid in our results supports this idea.

Our results also showed that Dopaquinone was significantly reduced in *C*Lso-infected PoP compared to the control. Dopaquinone is an oxidation product of dopamine under the action of tyrosinase (a phenol oxidase), which can be re-oxidized and deposited as melanin. Previous research has found that phenol oxidase (PO) plays an important role in the insect’s melanization defense response to invaders (Marmaras et al., 1996). Therefore, we concluded that the significant reduction of Dopaquinone is related to a reduced insect defense response, which facilitates the spread of psyllids to plants.

Ferulic acid (FA) is a type of active ingredient of phenolic acid that is derived from the dehydrogenation of ferulic acid glucoside and is involved in the formation and stabilization of plant lignin (Wang Y. L. et al., 2022). It also acts as an antioxidant, showing significant protective effects during cellular or tissue damage by neutralizing free radicals and reducing the formation of reactive oxygen species (ROS) (Liu et al., 2019). It has been reported that invasive pathogenic bacteria can cause systemic resistance in plants by increasing lignin and the antioxidant function of superoxide dismutase (SOD) to resist invasion (Ahmadifar et al., 2019). FA can inhibit the growth of microbes such as bacteria, protozoa, and fungi (Zuhainis Saad et al., 2008). However, some microbes have the ability to detoxify FA or even use FA as a single carbon source. For example, Zymomonas mobilis ZM4 can detoxify phenolic aldehydes (Yi et al., 2015).

In this study, increased levels of ferulic acid (FA) were found in *C*Lso-infected PoP. However, the presence of FA in insects or bacteria has yet to be identified, even though bacteria plays an important role in the release of FA. The successful colonization of plants by pathogens require an efficient utilization of nutrient resources present in host tissues; therefore, it is not surprising that hosts and pathogens have evolved distinct strategies to compete for this essential element. It can be speculated that the detected FA in PoP comes from the host, triggered by the *C*Lso pathogens transmitted when *C*Lso-infected psyllids feed on potatoes. Whether the FA can inhibit the growth of microbes or whether *C*Lso can use FA as a carbon source requires further research.

Prostaglandins (PGs) are effective immune mediators that can activate the immune response of insects to reject harmful pathogen infections. The effects of eicosanoids in the intimate relationship between host and pathogen have been observed in entomopathogenic nematodes (*Steinernema feltiae*) in lepidopteran insects and in a pathogenic bacterium, *Serratia marcescens*, in mosquitoes (Ahmed et al., 2022; Roy et al., 2022).

**Figure 5:**
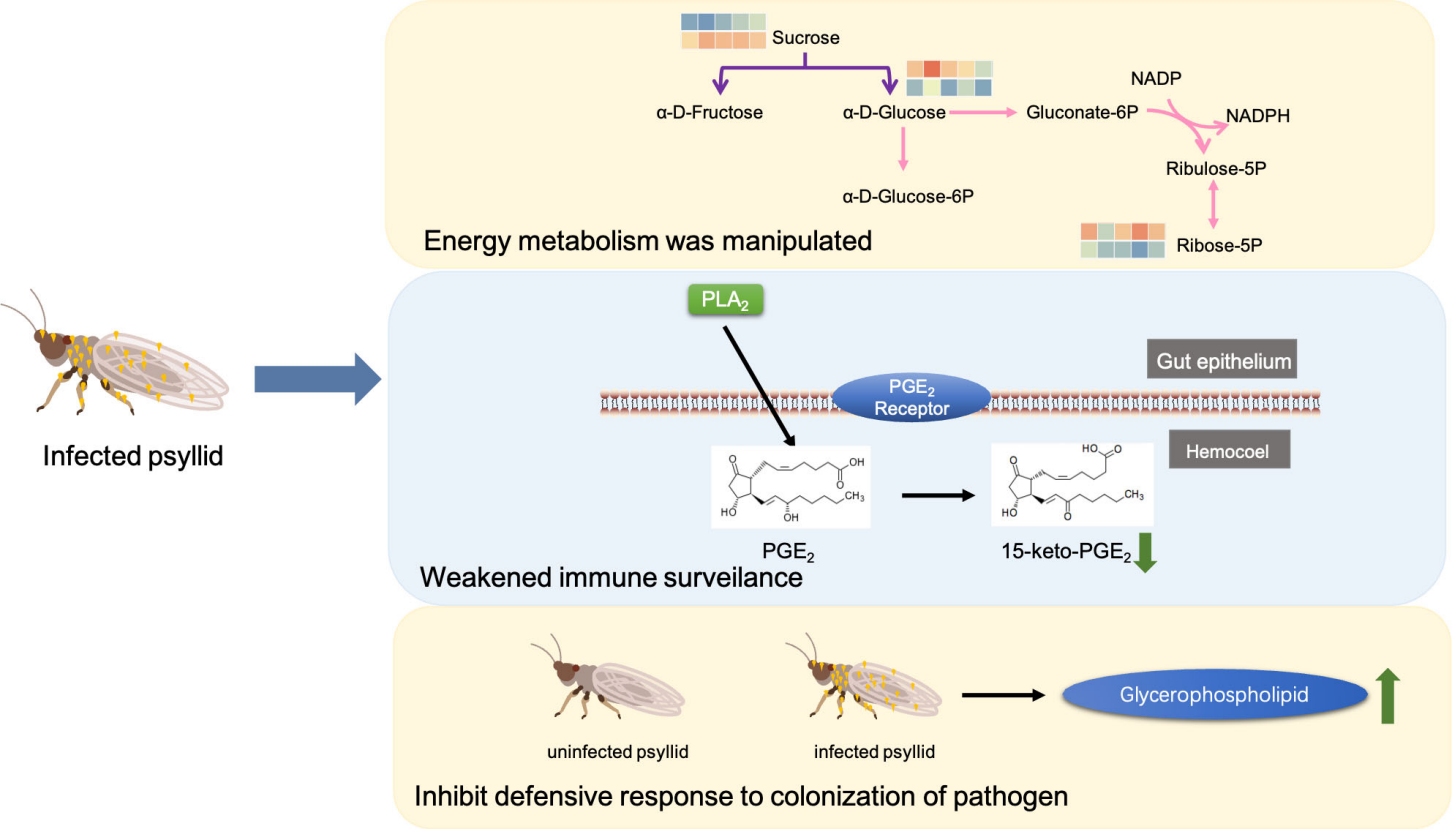
Schematic of possible mechanisms of *C*Lso infection in PoP.

Prostaglandin E2 (PGE2) is produced through the oxidation reaction of arachidonic acid (AA) and other polyunsaturated fatty acids, catalyzed by phospholipase A2 (PLA2) through biomembrane phospholipids under various physiological and pathological stimuli (Stanley, 2006; Burke and Dennis, 2009; Stanley and Kim, 2014). During bacterial infections, PGE2 is an extremely active lipid mediator in the immune response, helping the host through various mechanisms. However, it has also been reported that PGE2 can potentiate the survival of intracellular pathogens in the host. The down-regulation of PGE2 metabolites in psyllid midguts suggests that certain pathogens may escape immune surveillance by suppressing the host’s immune system and using its biological machinery to spread (**Figure 5**).

As prokaryotes, bacteria needs to acquire substrates such as cholesterol and sphingolipids from their eukaryotic hosts due to the lack of cellular synthesis tools. Not only are host lipids hijacked, but their metabolism is also altered during infection. Lipids play important roles in the interaction between hosts and pathogenic microorganisms. For example, they can serve as a potential carbon source for oxidative metabolism to restructure complex lipids (Poudyal and Paul, 2022), as mediators to regulate immune and inflammatory responses (Barletta et al., 2016), and as inhibitors of host immune defenses to promote self-colonization and development (Koga et al., 2010). Notably, pathogenic bacteria can inhibit host defense responses to colonization by increasing the lipid content of host macrophages (Koga et al., 2010).

For example, *Mycobacterium bovis* (MTB) infection mainly induces an increase in glycerophospholipids and polyketoic acid in macrophages, leading to the absorption of a large amount of lipids and the formation of foam cells. This in turn inhibits the defense function of macrophages and promotes their colonization and development (Gao et al., 2022). Phosphatidylcholine (PC) (16:1(9E)/0:0) is one of the biomarkers that causes changes in lipid metabolism. In this study, PC (16:1(9E)/0:0) was significantly decreased in *C*Lso-infected PoP. In addition, there was a significant increase in glycerophospholipids in *C*Lso-infected psyllids, such as PA(18:4(6Z,9Z,12Z,15Z)/18:4(6Z,9Z,12Z,15Z)), TG(12:0/12:0/20:5(5Z,8Z,11Z,14Z,17Z)), PC(12:0/18:1(9Z)), PE(12:0/18:3(6Z,9Z,12Z)), PC(O-16:0/22:4(7Z,10Z,13Z,16Z)), PE(16:0/18:1(9Z)). It is hypothesized that the changes in lipid metabolism in psyllids were caused by *C*Lso infection. *C*Lso secretes glycerophospholipids in psyllids to inhibit phagocytosis and clearance by the host insect, thus escaping the host’s immune defense response and achieving colonization. This study shows that pathogens can use specific strategies to modulate host lipid metabolism and homeostasis in order to maintain fluidity, survival, and dissemination.

The data generated by UPLC-MS can reveal small changes in the metabolic level of a sample and provide insight into the overall metabolic state of the organism. Meanwhile, MALDI-MSI is a reflection of spatial information, and can detect not only the molecules of interest, but also a large number of other analytes simultaneously. We found several organ-specific quality signals in psyllids. For example, a strong signal at *m/z* 416.138 (Ferulic acid), 723.365 (PA(18:4(6Z,9Z,12Z,15Z)/18:4(6Z,9Z,12Z,15Z))), and 644.030 (UDP-N-acetyl-alpha-D-glucosamine) was detected only below the abdomen of infected PoP (**Figure 4**, 1, 9, 10). 15-keto-Prostaglandin E2, Ornithine, PA(O-18:0/18:3(6Z,9Z,12Z)), PC(16:1(9E)/0:0), Dopaquinone, D-Ribose 5P, Octadecyl trichloroacetate, and α-D-Glucose were mainly expressed in the head and abdomen of uninfected PoP (**Figure 4**, 7, 8, 14, 15, 16, 22, 23, 26). The concentrations of these substances significantly differ in the insect.

The disadvantage of conventional metabolic analysis is that it is not achievable to explore the cell-to-cell heterogeneity in the context of an organ or tissue, while the emergence of MALDI-MSI overcomes this problem (Wang G. et al., 2022). MALDI-MSI as a method to measure the spatial distribution of metabolites at single-cell resolution, provides the possibility to understand the tissue-type-specific metabolic diversity of infected psyllid. According to recent studies, Benkacimi et al. successfully identified two species of bed bugs to the species level using MALDI-TOF/MS (Benkacimi et al., 2020). Tuthill et al. used a combination of HPLC-MS/MS and MALDI-MSI to spatially and temporally localize lipids in the heart and hemolymph of *Drosophila*, identifying potential endocrine mechanisms involved in lipotoxicity and metabolic diseases (Tuthill et al., 2020). These studies give us confidence and experience in studying the smaller insect in question. In this study, we first identified 34 different ions using untargeted UPLC-MS, and then examined the distribution of these candidate markers in PoP using MALDI-MSI imaging of insect tissues. These potential biomarkers can be used to quickly track *C*Lso and further understand the interaction mechanism between the psyllid and *C*Lso pathogens. This study may provides new insights into metabolic changes following *C*Lso infection of potato psyllids.

In conclusion, our results suggested that *C*Lso infection induces nutrient and energy stress, causing changes in the lipid metabolism of psyllids. This weakening of the insect vector’s immune defense response promotes colonization and transmission, which will help us better understand the interaction between psyllid and *C*Lso.

## Data availability statement

The original contributions presented in the study are included in the article/**Supplementary Material**. Further inquiries can be directed to the corresponding authors.

## Ethics statement

The animal study was reviewed and approved by The Special Committee of Science Ethics of Guangzhou University.

## Author contributions

YL: Provided data visualization, validation and wrote the manuscript. ZT: Data Analysis. LH: Provided data visualization, critically reviewed and revised the paper. XW: Formal Analysis, wrote original draft, writing-review and editing. All authors contributed to the article and approved the submitted version.
